# Raman spectroscopy accurately differentiates mucosal healing from non-healing and biochemical changes following biological therapy in inflammatory bowel disease

**DOI:** 10.1371/journal.pone.0252210

**Published:** 2021-06-02

**Authors:** Samuel C. L. Smith, Carl Banbury, Davide Zardo, Rosanna Cannatelli, Olga M. Nardone, Uday N. Shivaji, Subrata Ghosh, Pola Goldberg Oppenheimer, Marietta Iacucci

**Affiliations:** 1 Institute of Immunology and Immunotherapy, University of Birmingham, Birmingham, United Kingdom; 2 Chemical Engineering, University of Birmingham, Birmingham, United Kingdom; 3 University Hospitals Birmingham NHS Foundation Trust, Queen Elizabeth Hospital Birmingham, Birmingham, United Kingdom; 4 National Institute for Health Research (NIHR) Birmingham Biomedical Research Centre, University of Birmingham and University Hospitals Birmingham NHS Foundation Trust, Birmingham, United Kingdom; University of Illinois at Chicago, UNITED STATES

## Abstract

**Background:**

Mucosal healing (MH) is a key treatment target in the management of inflammatory bowel disease (IBD) and is defined in endoscopic terms by the newly published PICaSSO score. Raman Spectroscopy (RS) is based on the scattering of inelastic light giving spectra that are highly specific for individual molecules. We aimed to establish spectral changes before and after treatment and whether Raman Spectroscopy is able to accurately differentiate between inflammation and MH.

**Methods:**

Biopsies were taken for *ex vivo* RS analysis alongside biopsies for histological analysis from IBD patients undergoing optical diagnosis endoscopic assessment. We compared pre- vs. post-biological treatment in IBD patients and healthy controls and active vs. MH in UC and CD. For spectral analysis, we used supervised self-organising maps for separation and classification.

**Results:**

A total of 23 patients (14 IBD, 9 HC) were recruited for comparison of pre- vs. post-biologic treatment and 74 IBD patients were included for the assessment of MH in IBD, giving 9700 Raman Spectra. Spectral differences were seen between pre- and post-treatment which were observed comparing MH vs. active inflammation. Reductions in intensity at 1003cm^-1^ and 1252cm^-1^ when a reduction in inflammation was seen post-treatment and when MH was present. MH was associated with an increase in intensity at 1304cm^-1^. The trained neural network differentiated MH from active inflammation with a sensitivity, specificity, PPV, NPV and accuracy in UC of 96.29% (sd 0.94), 95.03% (sd 1.52), 94.89% (sd 1.59), 96.33 (sd 0.97) and 95.65 (sd 0.99) and 96.19% (sd 1.46), 88% (sd 4.20), 86.60% (sd 5.39), 96.55% (sd 1.32) and 91.6% (sd 2.75) in CD respectively.

**Conclusion:**

We demonstrated RS can demonstrate biochemical changes following treatment of IBD and accurately differentiates MH from active inflammation in IBD and might be a future tool to personalise therapeutic management in IBD.

## Introduction

The current recommended treatment strategy of inflammatory bowel disease (IBD) is “treat to target”, whereby clinicians aim to achieve clearly defined outcomes [1, 2]. A key target is that of mucosal healing (MH) [1, 2], since clinical remission in isolation is no longer considered a reliable measure of response to therapy and prognosis. MH is associated with improved patient outcomes with fewer episodes of hospitalisation, surgical intervention and corticosteroids [3].

However, MH is poorly defined, the assessment of which is open to debate. Endoscopic remission has traditionally been used to define MH, but inflammatory activity at histologic level can remain [4]. It is increasingly recognised that histological remission (HR) also predicts better outcomes [5, 6], therefore definitions of MH is starting to incorporate endoscopic and histological remission. The recently published PICaSSO (The Paddington International Virtual Chromoendoscopy Score) score was developed to better define subtle mucosal and vascular changes of UC and for the first time introduced endoscopic findings of MH by using virtual electronic chromoendoscopy (VEC) [7–9]. This is an evolving field, which enables endoscopic assessments of subtle inflammatory changes.

Despite apparent endoscopic MH (Mayo Endoscopic Score (MES) 0) [10], up to 9.4% of patients relapse within 6 months [11]. It is likely that there are subtle molecular changes present in those patients that suffer early relapse, which have yet to manifest at the endoscopic level. Raman Spectroscopy (RS) is one technology that describes the molecular constituents of biological tissue and is the phenomenon when light strikes a tissue causing vibrational energy change of individual molecules [12]. The frequency difference between incident and scattered light characterises the molecule vibration, which are indicative of chemical bonds or groups of bonds which is manifested in the form of a spectrum highly specific for individual molecules thereby giving a “biomolecular fingerprint” allowing immediate analysis of the “make-up” of biochemical tissue. Its application in differentiating CD and healthy control with an accuracy of 83.6% using plasma has been demonstrated recently [13]. The potential of subtle biomolecular signals of low-grade inflammation before mucosal defects appear, to predict relapse is a key objective but the threshold at which subtle inflammation can be detected has first to be established. However, a consistent drawback of RS is that, due to its weak signal, the signal is complex making analysis challenging [14]. Traditional methods of analysing RS data relied upon PCA (principal component analysis), which has several limitations [15]. A novel method utilising self-organising maps, the Self-Optimising Kohonen Index Network (SKiNET), was developed which has shown promising results in studies involving eye tissue [14] and has the potential to revolutionise the clinical application of RS.

The aim of this pilot study was by using a multi-step process and using SKiNET, for the first time assess the ability of RS to differentiate between active IBD and MH defined in histo-endoscopic terms and establish spectral changes following successful biological treatment.

## Materials and methods

### Patients

Patients with IBD were prospectively enrolled in a non-selective manner meeting inclusion criterion below from a single tertiary centre, University Hospitals Birmingham NHS Foundation Trust between August 2018 and October 2019, who underwent colonoscopy. In order to better define spectral changes associated with MH a sub-group of patients who underwent colonoscopy before and 12–16 weeks following biological therapy were also included. Inclusion criteria included a proven diagnosis of IBD and age 18–80. Exclusion criteria included inability to consent, coagulopathy and pregnancy/breast-feeding. Finally, patients without IBD who underwent colonoscopy for non-IBD polyp surveillance were recruited to provide a healthy control group and were excluded if macroscopic or microscopic inflammation was present.

### Endoscopic procedure

**Fig 1:**
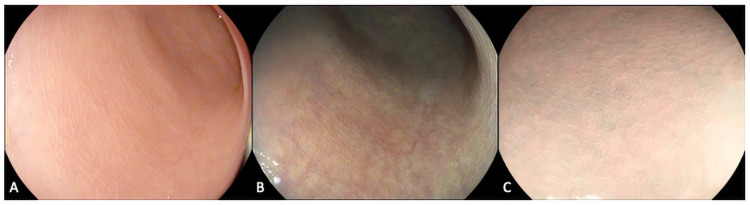
Endoscopic assessment of IBD. Colonic mucosa was examined using high-definition white light and virtual electronic chromoendoscopy demonstrating vascular and mucosal architecture of healing using a) iScan 1 b) iScan 2 c) iScan Optical Enhancement.

All procedures were performed by an endoscopist (MI) experienced in optical diagnosis and IBD using Pentax 7010 processor (Pentax, Tokyo, Japan) and Olympus 290 series endoscopes (Olympus, Tokyo, Japan). Colonic mucosa was inspected, and precise assessments of inflammatory activity took place using high-definition white light endoscopy and Virtual Electronic Chromoendoscopy (Optical Enhancement iScan mode/Narrow-Band Imaging (NBI)) with and without magnification (Fig 1A–1C). Inflammatory activity was graded using the MES [10], Ulcerative Colitis Endoscopic Index of Severity (UCEIS) [16] and the PICaSSO score [7, 9] in patients with UC and SES-CD in patients with CD [17]. Using these advanced endoscopic technologies and novel scores such as the PICaSSO score [9], targeted biopsies of inflamed/healed areas, which best reflected the overall inflammatory activity irrespective of colonic segment, were taken for *ex vivo* RS analysis and histological analysis at the same site. One site per patient was selected. Biopsies taken for RS were placed in an empty aliquot and immediately snap frozen in liquid nitrogen and kept at -80°C.

### Study definitions

Response to biological therapy was described as a reduction in endoscopic inflammatory activity score and in histological inflammatory score. The following stringent criteria were used to define patients with MH: MES [10] ≤1, UCEIS (Ulcerative colitis endoscopic index of severity) score [16] ≤1, PICaSSO score [7, 9] ≤3 in addition to histological remission defined as RHI (Robarts Histological Index) score ≤3 without neutrophils in the epithelium and Lamina Propria [18] for UC. The following criteria were used to define MH in CD patients: SES-CD (Simple endoscopic score for Crohn’s disease) [17] ≤2 and a modified Riley score 0 in CD [19]. Patients who did not meet these criteria were classified as having active inflammation.

### Raman spectroscopy measurement protocol

**Fig 2:**
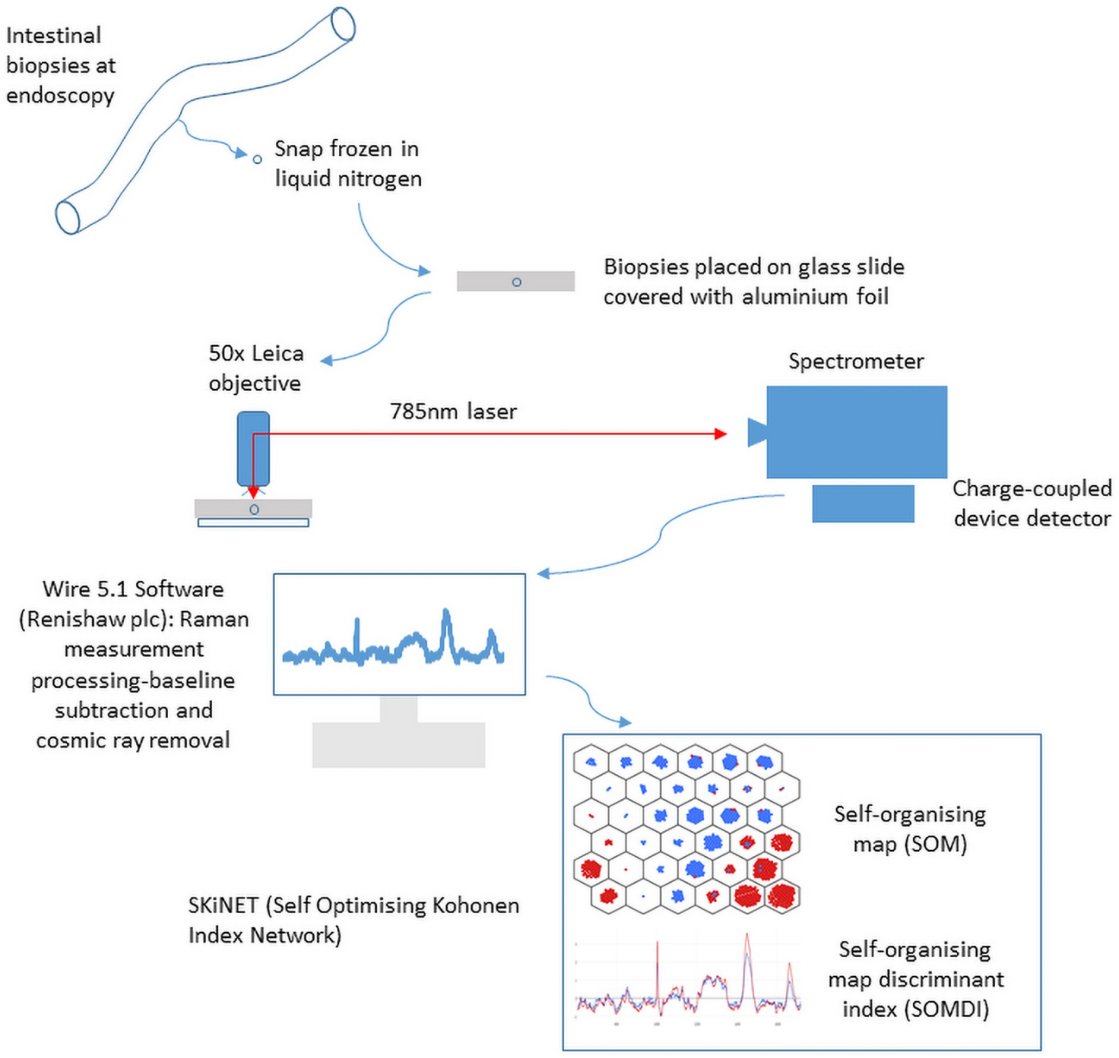
Overview diagram demonstrating the process of sample acquisition and preparation for Raman Spectral measurement and data analysis.

Biopsies were placed on aluminium foil covered glass slides and analysed at room temperature. Measurements were collected using an InVia Qontor (Renishaw plc, Wotton-under-Edge, UK) with a 785nm (10mW) laser, 1s acquisition, 10 accumulations for each point on a 10x10 surface map scan, resulting in 100 spectra per patient. The spectrometer was calibrated prior to each use with a silicon wafer (520.7cm^-1^). A 50x Leica objective, 1200l/mm grating were used for all scans in the range 550-1670cm^-1^. Wire 5.1 (Renishaw plc) software was used to process the measurements, subtract baseline and remove cosmic rays to all spectra. Fig 2 illustrates the process of RS and data analysis.

### Self-organising maps and self optimising Kohonen Index Network

The RS measurement protocol was completed prospectively following patient enrollment. At the end of recruitment, artificial neural networks (Self-organising maps-SOM) and a supervised learning model were developed to demonstrate spectral differences and build predictive modelling [14]. A class was defined as a group of data to identify and discriminate, for example: MH vs. active inflammation and pre-biologic vs. post-biologic. To define a classification model, we use a pool of training data where we know the class of the data to “teach” a machine (80% of data), which is pulled at random. This process has been established and previously published [14]. The model was validated by presenting test data (20% of data) that the model has not seen before. RS is multi-dimensional and has a high degree of intra- and inter-class variability for complex samples, such as tissue [20]. The Self-organising map (SOM) is a method that can reduce the hyper-dimensional nature of RS to a two-dimensional model. A SOM represents an artificial neural network arranged as a map of hexagonal neurons [21]. Each neuron activates for certain inputs (spectra) and not others. Neurons that activate on similar inputs to their neighbours, and therefore clustering occurs when multiple neurons group together when activated by the same characteristic, similar to a human brain [14]. A self-organising map discriminant index (SOMDI) [14] is extracted from this process, which allows us to understand which features of the training data causes the spatial clustering and separation of classes observed in the SOM. The magnitude in the SOMDI relates to how strongly a variable is considered a discriminator for a given class. This results in a representation similar to a spectrum, where we can compare SOMDI for each group, and identify Raman bands that are more important to one class from another. We exploited the newly developed SKiNET (Self Optimising Kohonen Index Network) approach, which provides a means of supervised learning to further optimise the result [14].

### Statistical analysis

The model was optimised by 10-fold stratified cross-validation and oversampling to account for class imbalance in the training data. Since the present study was a pilot study, using for the first time SKiNET [14] in IBD, a sample size calculation was not possible. In order to further confirm the difference between classes we ran the analysis 10 times and took an average sensitivity, specificity, PPV, NPV and accuracy plus standard deviation.

### Ethical approval

Ethical approval was granted by the Office for Research Ethics Committees Northern Ireland (17/NI/0148) and the South West-Frenchay Research Ethics Committee (19/SW/0010). Written informed consent was obtained for all participants.

## Results

### Assessment of mucosal healing in IBD

A total of 74 patients were included (42 UC and 32 CD) (Table 1) with 7400 Raman Spectra acquired overall. The median age of patients recruited was 41.2 years (± 13.4, range 19–66), and 34 (45.9%) of patients were male.

**Table 1:** Demographics of patients included in the analysis of mucosal healing vs. active inflammation.

Characteristics	Patients
Age, median ± sd (yrs)	41.2 ± 13.4
Gender	
Male (%)	34 (45.9%)
Female (%)	40 (54.1%)
IBD type	
UC (%)	42 (56.8%)
CD (%)	32 (43.2%)
Therapy, n (%)	
UC	
NSAIDs	6 (14.3%)
5-ASA	37 (88.1%)
Steroids	26 (61.9%)
Thiopurine	28 (66.7%)
Biologic	15 (35.7%)
CD	
NSAIDs	6 (18.8%)
5-ASA	5 (15.6%)
Steroids	18 (56.3%)
Thiopurine	22 (68.8%)
Biologic	17 (53.1%)

#### Active inflammation vs. MH in UC

**Fig 3:**
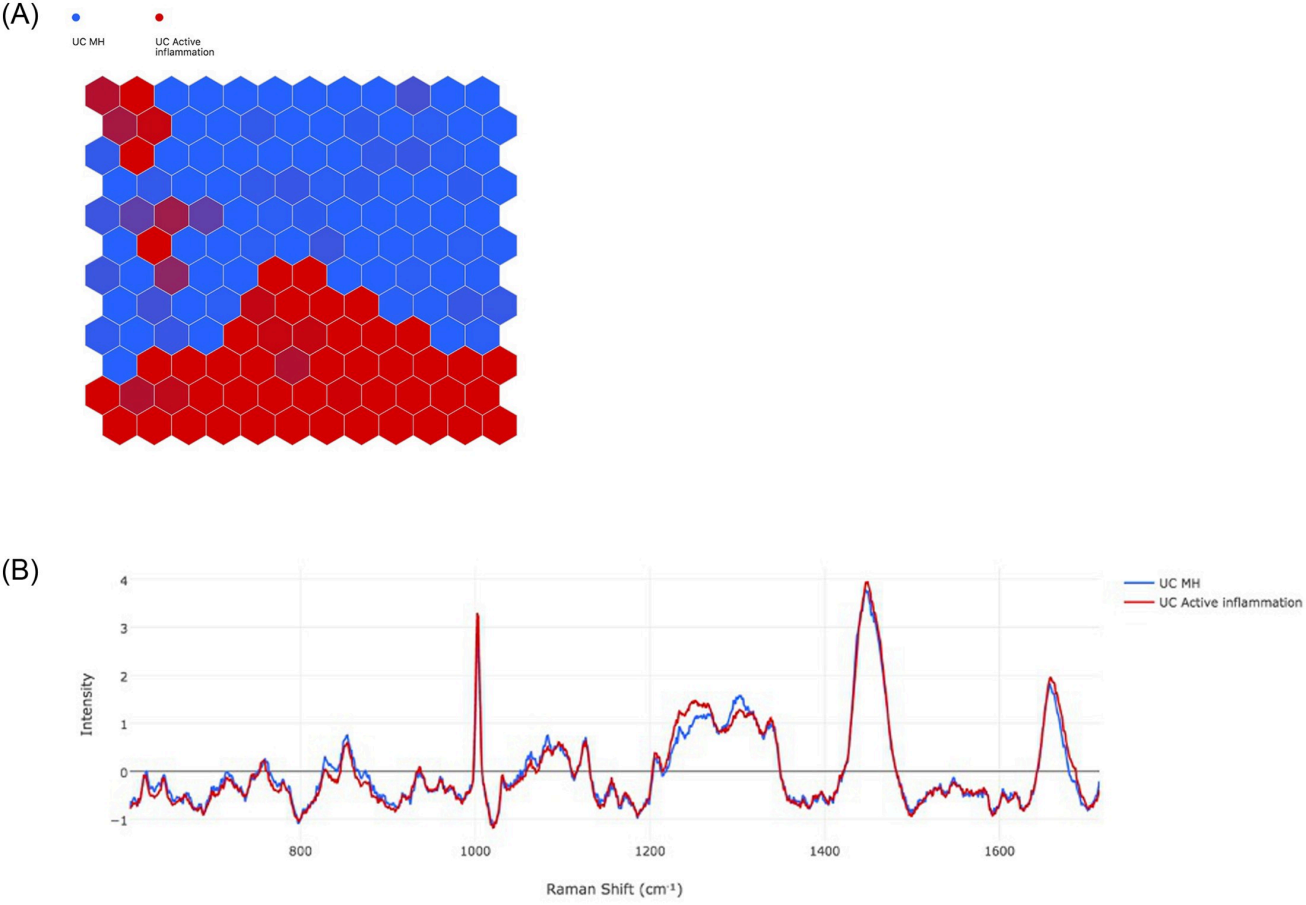
UC active inflammation (red) vs. UC mucosal healing (blue). a) Self-organising Map (SOM) demonstrates the grouping. Each dot on the map represents a neuron, and each neuron represents a spectrum. Each new neuron will be drawn to its nearest vector, thereby causing clustering of neurons that have similar characteristics. The colour of the hexagon represents the heaviest weighting of neurons b) Self-organising Map Discriminant Index (SOMDI) providing a visual representation of the SOM data, highlighting the differences in spectra.

A total of nine (21.4%) UC patients had evidence of MH whilst 33 (78.6%) had active inflammation. Fig 3a demonstrates the SOM, showing the MH (blue) vs. active inflammation (red) groups are well separated. The key spectral differences from the SOMDI (Fig 3b) are presented in Table 2 (cross-validation accuracy 0.95). The trained artificial neural network was able to differentiate between MH and active inflammation in 20% of the testing dataset with a sensitivity, specificity, positive predictive value (PPV), negative predictive value (NPV) and accuracy of 96.29% (sd 0.94), 95.03% (sd 1.52), 94.89% (sd 1.59), 96.33 (sd 0.97) and 95.65 (sd 0.99) respectively (S1 Table).

**Table 2:** Relative intensity of Raman shifts between UC MH vs active inflammation taken from SOMDI.

Raman Shift (cm^-1^)	UC Active inflammation (Intensity)	UC Mucosal Healing (Intensity)
1003	3.30	2.69
1252	1.44	1.11
1304	1.27	1.54
1449	3.92	3.73
1657	1.93	1.83

#### Active inflammation vs. MH in CD

**Fig 4:**
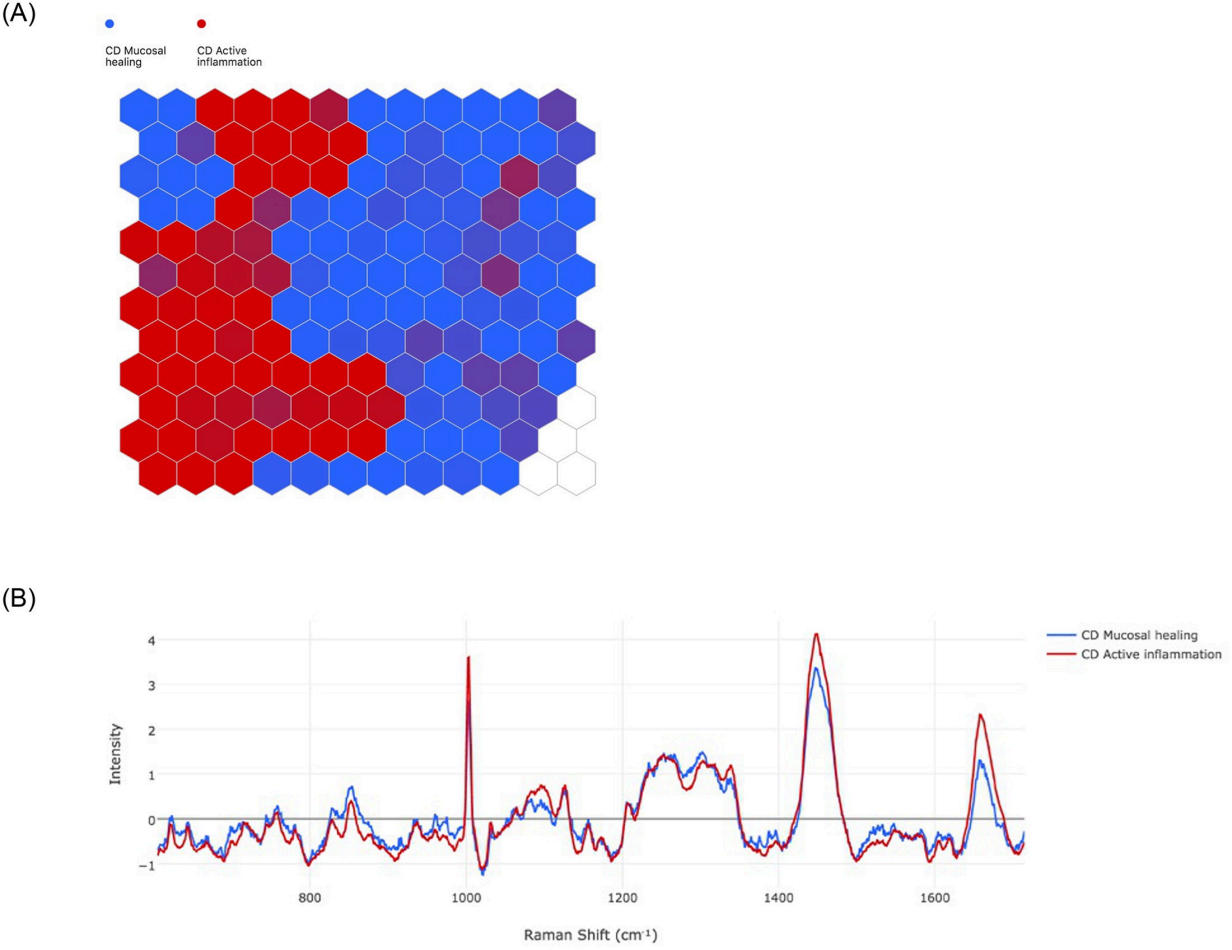
CD active inflammation (red) vs. MH (blue). a) SOM demonstrates the groupings of MH vs active inflammation and b) SOMDI highlighting the spectral differences from the SOM.

A total of ten (31.3%) CD patients had evidence of endoscopic and histological healing and 22 (68.8%) active inflammation. The separation between MH CD (blue) and active inflammation CD (red) is demonstrated by the SOM in Fig 4a. The spectral differences presented in SOMDI (Fig 4b) are demonstrated in Table 3. The artificial neural network was able to differentiate the remaining 20% of the data with a sensitivity, specificity, PPV, NPV and accuracy of 96.19% (sd 1.46), 88% (sd 4.20), 86.60% (sd 5.39), 96.55% (sd 1.32) and 91.6% (sd 2.75) respectively (S2 Table).

**Table 3:** Relative intensity of Raman shifts between CD healing vs non-healing taken from SOMDI.

Raman Shift (cm^-1^)	CD Active inflammation (Intensity)	CD Mucosal Healing (Intensity)
1003	3.60	2.63
1252	1.40	1.40
1304	1.30	1.46
1449	4.11	3.30
1657	2.32	1.30

### Biochemical changes following response to biological therapy

A total of 23 patients were included (6 UC, 8CD and 9 HC) in the exploratory analysis comparing spectral features in patients with IBD before and after biological therapy with patients without IBD (Table 4). The median age of patients in this analysis was 44.0 (+/- 14.8, range 25–79), nine were male (39.1%) and all UC patients all but one CD patients (7 out of 8) were biologic naïve.

**Table 4:** Demographics of patients included in the exploratory analysis of pre- vs. post-biologic therapy and patients without IBD (healthy control).

Characteristics	Patients
Age, median ± sd (yrs)	44.0 ± 14.8
Gender	
Male (%)	9 (39.1%)
Female (%)	14 (60.9%)
IBD type	
UC (%)	6 (26.1%)
CD (%)	8 (34.8%)
Non-IBD	9 (39.1%)
Therapy, n (%)	
UC	
NSAIDs	1 (16.7%)
5-ASA	6 (100.0%)
Steroids	4 (66.7%)
Thiopurine	3 (50.0%)
Biologic	0 (0.0%)
CD	
NSAIDs	1 (12.5%)
5-ASA	1 (12.5%)
Steroids	6 (75.0%)
Thiopurine	7 (87.5%)
Biologic	1 (12.5%)

#### Pre- vs. post-treatment in UC and healthy controls

**Fig 5:**
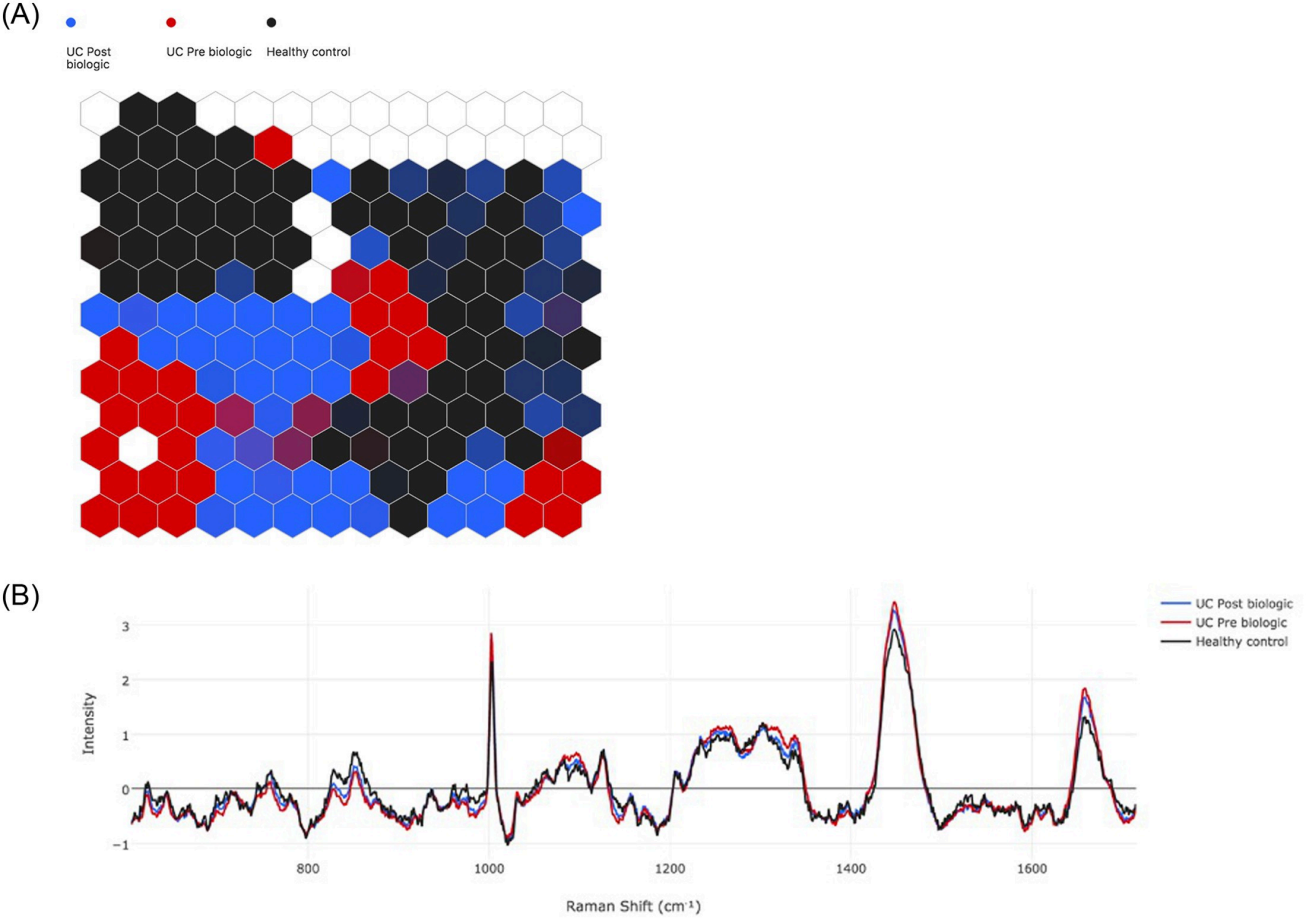
UC pre- (red) vs. post-biologic therapy (blue) vs. healthy control (black). a) Self-organising Map (SOM) b) Self-organising Map Discriminant Index (SOMDI) highlighting the spectral differences from the SOM.

The separation between pre- (red) vs. post-biologic (blue) in UC patients and healthy control (black) is shown by the SOM in Fig 5a and the spectral differences are shown in the SOMDI in Fig 5b. The key spectral differences that differentiate the 3 groups are presented in Table 5 (cross-validation 0.95).

**Table 5:** Relative intensity of Raman shifts between UC pre biologic and post biologic and HC taken from SOMDI.

Raman Shift (cm-1)	UC Pre Biologic (Intensity)	UC Post Biologic (Intensity)	Healthy control (Intensity)
1003	2.85	2.72	2.32
1252	1.13	1.04	0.99
1304	1.17	1.10	1.16
1449	3.42	3.24	2.91
1657	1.84	1.68	1.32

#### Pre- vs. post-treatment in CD and healthy controls

**Fig 6:**
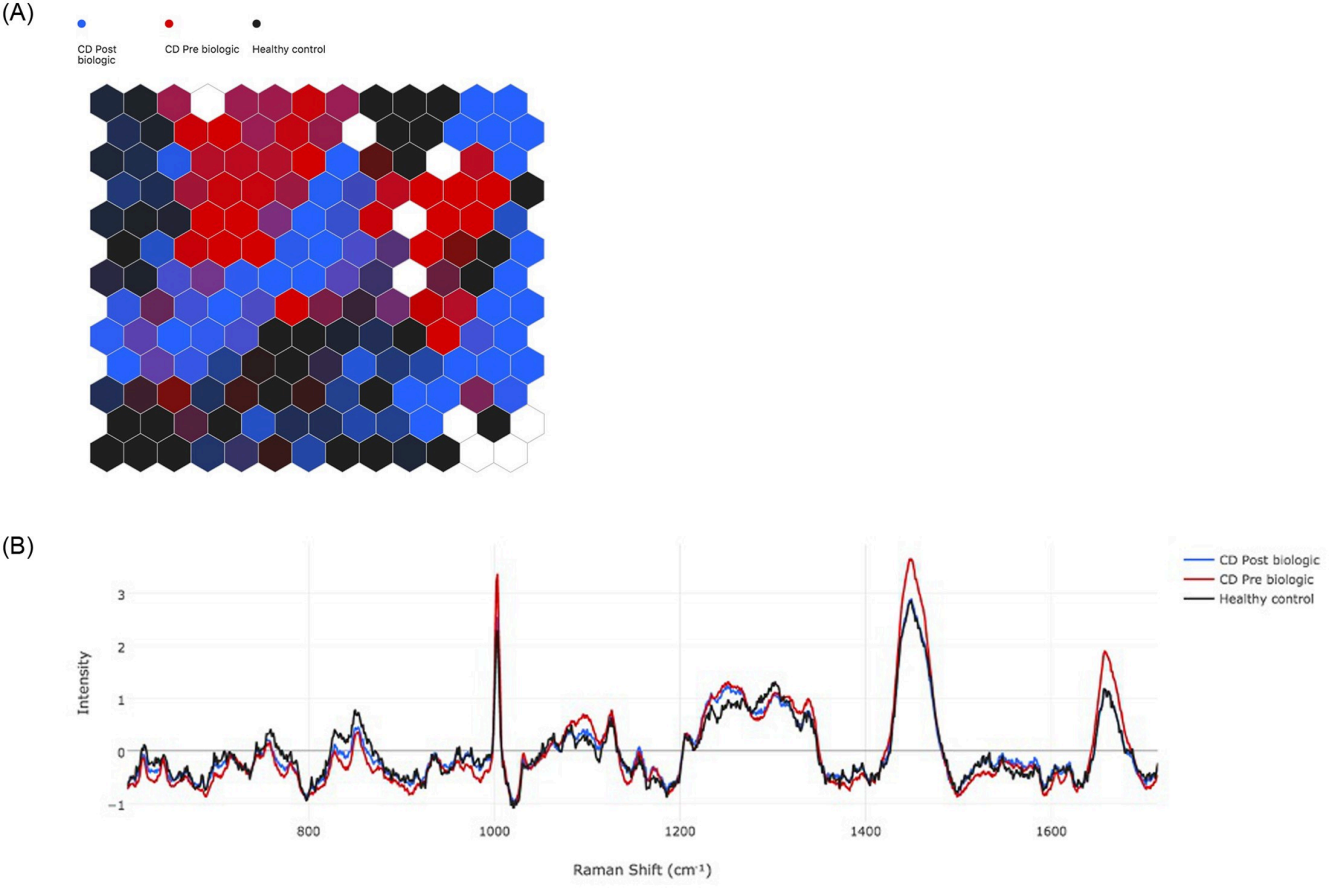
CD pre- (red) vs. post-biologic therapy (blue) vs. healthy control (black). a) Self-organising Map (SOM) demonstrates the groupings and b) Self-organising Map Discriminant Index (SOMDI) highlights the spectral differences from the SOM.

The separation of pre-biologic CD (red), post-biologic CD (blue) and HC (black) is presented in the SOM in Fig 6a, and SOMDI demonstrating the spectral features is shown in Fig 6b (cross-validation 0.83). The spectral differences that differentiate the groups taken from the SOMDI are presented in Table 6. This exploratory analysis provided us information on the spectral features to focus on in the differentiation between MH and active inflammation in IBD.

**Table 6:** Relative intensity of Raman shifts between CD pre-biologic and post-biologic and HC taken from SOMDI.

Raman Shift (cm-1)	CD Pre Biologic	CD Post Biologic	Healthy control
1003	3.38	2.49	2.29
1252	1.32	1.24	0.98
1304	1.10	1.05	1.27
1449	3.66	2.90	2.87
1657	1.89	1.84	1.17

### Biomolecular findings

The Raman band at 1003cm^-1^ is associated with the amino acid phenylalanine [22, 23], and our results show a relative reduction at this band in MH and when there is a reduction in inflammatory activity and a lower level in healthy controls. We found a higher intensity at Raman band 1252cm^-1^ in UC when inflammation is present compared with MH, which may represent amide III [22–24] and lower intensity was seen in healthy controls. An increase in intensity at 1304cm^-1^, seen in both UC and CD when MH is present, likely corresponds to phospholipids [23, 25, 26]. A reduction in intensity at 1657cm^-1^ in UC and CD patients in MH, following treatment with biological therapy and healthy controls which could be attributed to C = C stretching in lipids [23, 26, 27].

## Discussion

An emerging key target in IBD is MH given the association with favourable outcomes for patients [1, 2]. Endoscopic assessments of MH can be inaccurate, particularly using older scoring systems such as the MES [4]. Using a multi-step process and using novel spectral analysis software, we established Raman spectral changes before and after biological therapy in IBD for the first time and compared MH vs. active inflammation in UC and CD. We have identified relative Raman spectral changes in keeping with MH in CD and UC, defined in histological and endoscopic terms-so-called endo-histological MH. We demonstrated that an artificial neural network is able to differentiate between MH and active disease with high accuracy in both UC and CD.

The discrepancy between endoscopic and histological assessment of MH is subject of considerable controversy. Significant proportions of patients who appear in endoscopic remission still have evidence of microscopic inflammation and suffer early relapse [28]. Whilst histological assessment of inflammation is considered gold standard and may help predict prognosis [29–31], it is reliant upon accurate biopsy sampling and incurs significant healthcare costs. RS has been emerging as a candidate technique to provide the crucial missing information since it is known to be able to give a rapid assessment of the biochemical “make-up” of biological molecular tissue. The *in vivo* use could provide optical diagnoses without the need for biopsies [26, 27], which would be safer for patients as well as offering significant cost benefits. RS does not require specialist tissue preparation, labelling, is non-destructive and provides an overview of biochemical composition rather than an individual biomarker [32, 33].

The main aim of this study was to explore the most important differences in spectra between MH and active inflammation. Therefore, we focused on the tallest peaks, since the higher the peak, the more important the corresponding wavelength is in differentiating the classes. The RS distribution were very similar between CD, UC and HC, but subtle differences in intensities of various Raman bands is noted between inflammation and healing, which is consistent with previous work [13].

The reduction in Raman band at 1003cm^-1^, which is associated with the amino acid phenylalanine [22, 23], in MH and following successful treatment with biological therapy is consistent with previous work by *Addis et al*. [34]. We found increases at Raman band 1003cm^-1^ when inflammation is present in both UC and CD. Higher levels of Phenylalanine has been reported to be associated with immune activation and inflammation [35], therefore when there is active inflammation present in both UC and CD and pre-biological therapy treatment there are greater infiltration of inflammatory cells thereby increasing the quantity of phenylalanine, which is reflected in the results of this study. Likewise, when there is reduced inflammatory activity or healing present, there is less inflammatory cell infiltrate, as reflected by histological scores, thereby reducing the quantity of amino acids such as phenylalanine. The reduction at this band following biological therapy, brought intensities that were more in line with healthy control patients. The higher intensity at Raman band 1252cm^-1^, which is likely a constituent of haem [22, 24], is consistent with inflammatory activity [24]. However, this was not found when comparing active inflammation and MH directly in CD. This lack of consistency may reflect the fact that UC is a mucosal disease and is associated with bleeding when inflammation is present on mucosal surface, more so than CD. The increase in intensity at 1304cm^-1^, seen in both UC and CD when MH is present, likely corresponds to phospholipids, which are key to the integrity of the bowel wall [25, 26]. There was an increase at 1304cm^-1^, in both UC and CD, when MH was present, which as likely relates to phospholipids [23, 26]. This has potential clinical significance, as it demonstrates a potential positive biomarker suggesting MH is present. Given the limitations of endoscopic and histological evaluations of MH, this may provide an additional measure to confirm the presence of MH.

The hyper-spectral nature can make RS analysis challenging. Traditionally principal component analysis (PCA) has been used, however this has several limitations [15]. With the aid of SKiNET [14], subtle differences can be detected. This has a potential application in clinical practice when assessing response to treatment, particularly when considering de-escalation of biological therapy. Stopping therapy in IBD in order to minimise long term side effects and risks of treatments, the “Exit strategy”, is a hot topic in IBD management [36] and is a difficult decision for clinicians largely due to a lack of robust guidance [37]. The risk of relapse within 1 year of biological cessation is as high as 38–44% [38]. In those patients who are at risk of short-term relapse, it is probable that biochemical changes are present which have yet to manifest as mucosal changes, and RS could have a role in identifying those patients.

Early studies aiming to differentiate active from inactive UC give insight into the potential of RS. *Addis et al*. included 39 patients with UC and identified Raman band differences between active and healed UC however this study was limited by single scan measurements on each biopsy and by the use of the MES [10] as a definition of healing [34]. *Ding et al*. recruited 18 patients with UC and found a machine-learning algorithm is able to differentiate between inactive UC from inflamed UC with an area under the curve of 0.83 [26]. Whilst the healing patients were identified using the modified Riley score, there was no endoscopic scoring of inflammatory activity.

To our knowledge, we have demonstrated for the first time that RS can be highly accurate in differentiating between active inflammation and MH, using robust endoscopic and histological definitions, in both UC and CD. By including 97 patients (88 IBD, 9 HC), measuring 100 spectra per patient (giving 9900 spectra in total), the present study is the largest in terms of patient number and spectra in IBD [24, 26, 39]. This study is also the first to demonstrate spectral changes following treatment of IBD and compare MH and active inflammation in CD. A further strength of the present study is in using the latest optical enhancement endoscopic technology, we ensured accurate assessments of inflammatory activity. A limitation to the study is that results were acquired in the *ex vivo* setting, thereby laying an important foundation for further *in vivo* application. For this purpose, laser power, total acquisition time and auto fluorescence issues will need to be carefully considered. Nevertheless, our aim was to explore spectral differences, and a potentially more focused map scan only including those wavelengths of interest could take place *in vivo*, which would take less time. Each patient was represented by 100 spectral maps in this analysis. Ideally these 100 maps would be averaged and 1 spectra per patient would be analysed. However, large patient numbers would be required for this otherwise would be subject to bias from outlier patients. A further limitation is that one colonic segment and one RS biopsy was taken per patient, therefore we are unable to assess whether similar results of healing vs. active inflammation can be seen within the same patient if patchy inflammation is seen. Biopsies taken from mucosa in endoscopic remission which later had evidence of histological activity were included in the active inflammation groups. A further area of interest would be in identifying subtle spectral differences in patients with endoscopic MH but histological inflammation from patients with endo-histological remission, as this would help shape decision making in IBD.

Identifying fibrosis in CD, can be challenging, with regards to differentiating fibrotic from inflammatory strictures using existing assessment modalities. It is probable the biochemical make-up of fibrosis differs from inflammation and therefore RS could be a useful management adjunct. RS probes that can be used via endoscopy channels are being designed for colonoscopes and our study shows the basis for exploring further such probes for assessment of response to therapy and mucosal healing at a molecular level in IBD.

## Conclusion

Overall, we successfully established spectral changes before and after biological therapy in IBD and differentiated between active inflammation and histo-endoscopic MH in UC and CD using RS and SKiNET. RS may be able to give more detailed information to clinicians on the presence or absence of MH. For many years the focus of IBD management has been on achieving endoscopic MH, and in more recent years the role of histological healing is increasingly being recognised. This study provides insight into the potential role of an additional mode of healing: molecular healing, which could further ensure robust assessment of healing. Further multicentre studies in this field needs to take place before this can be used in everyday clinical practice.

## Supporting information

S1 Table Example confusion matrix of the trained predictive model at differentiating MH from active UC. (DOCX)

S2 Table Example confusion matrix of the trained predictive model at differentiating MH from active CD. (DOCX)

S1 Dataset Healthy control. (ZIP)

S2 Dataset Export UC MH. (ZIP)

S3 Dataset Export UC active inflammation. (ZIP)

S4 Dataset CD mucosal healing. (ZIP)

S5 Dataset CD active inflammation. (ZIP)

S6 Dataset Post biologic CD. (ZIP)

S7 Dataset Post-biologic. (ZIP)

S8 Dataset Pre biologic CD. (ZIP)

S9 Dataset Pre-biologic. (ZIP)

## Abbreviations

CD: Crohn’s disease
HR: Histological remission
IBD: Inflammatory bowel disease
IC: Indeterminate colitis
MES: Mayo Endoscopic Score
NBI: Narrow Band Imaging
OE: Optical enhancement
PICaSSO: The Paddington International Virtual Chromoendoscopy Score
SES-CD: Simple endoscopic score for Crohn’s disease
UC: Ulcerative colitis
UCEIS: Ulcerative colitis endoscopic index of severity
VEC: Virtual Electronic Chromoendoscopy
