# Supplementary material for: Raman spectroscopy accurately differentiates mucosal healing from non-healing and biochemical changes following biological therapy in inflammatory bowel disease
Source: PLoS One. 2021 Jun 2;16(6):e0252210. doi: 10.1371/journal.pone.0252210 (PMC8172032; doi:10.1371/journal.pone.0252210)
Supplement: S1 Table — (DOCX) [file pone.0252210.s001.docx]

S1 Table.

|  | UC MH | UC Active inflammation |
| --- | --- | --- |
| UC MH | 170 | 10 |
| UC Active inflammation | 7 | 173 |
