# Supplementary material for: Raman spectroscopy accurately differentiates mucosal healing from non-healing and biochemical changes following biological therapy in inflammatory bowel disease
Source: PLoS One. 2021 Jun 2;16(6):e0252210. doi: 10.1371/journal.pone.0252210 (PMC8172032; doi:10.1371/journal.pone.0252210)
Supplement: S2 Table — (DOCX) [file pone.0252210.s002.docx]

S2 Table.

|  | CD MH | CD Active inflammation |
| --- | --- | --- |
| CD MH | 181 | 19 |
| CD Active inflammation | 7 | 193 |
